# Enhancing motor coordination and social interaction in children with autism through virtual reality rehabilitation

**DOI:** 10.1097/MD.0000000000045950

**Published:** 2025-11-21

**Authors:** Hui Yi, Xiufang Zhou, Shengju Quan, Tianyan Liu

**Affiliations:** a Department of Pediatric, Ward 3, Affiliated People’s Hospital of Hubei Medical College, Shiyan, Hubei, China.

**Keywords:** autism, caregiver satisfaction, motor coordination, social interaction, virtual reality

## Abstract

This study evaluates the effects of virtual reality (VR) rehabilitation on motor coordination and social interaction in children with autism and explores related neural mechanisms and caregiver perspectives. A retrospective analysis was conducted on 80 children with autism who underwent either VR-based or conventional rehabilitation. Outcomes included motor performance, social behaviors, training adherence, caregiver satisfaction, and brain activation assessed by functional near-infrared spectroscopy. Compared with conventional training, VR rehabilitation was associated with greater improvements in motor coordination and social engagement. Enhanced activation was observed in brain regions linked to motor control and social cognition. Higher training adherence was positively correlated with caregiver satisfaction. VR-based rehabilitation can effectively support motor and social development in children with autism while improving treatment adherence and caregiver acceptance. These findings highlight VR as a promising tool for personalized pediatric neurorehabilitation.

## 1. Introduction

With the progress of science and technology, the use of virtual reality (VR) in neural rehabilitation has gradually expanded.^[1,2]^ VR technology can build a safe and interactive virtual environment, increase the interest and participation of patients in the training process, and promote the remodeling of patients’ neurological function.^[3,4]^ Recent clinical trials have confirmed that VR systems enhance cerebellar-basal ganglia circuit function through multi-sensory feedback mechanisms such as visual-vestibular integration, which is critical for motor learning.^[5,6]^ Autism spectrum disorder (ASD) is a neurodevelopmental disorder, which causes the children to have limb incoordination (such as 73% of children have abnormal gait cycle^[7]^) and social difficulties (only 32% can not talk for more than 3 rounds^[8]^). Long-term rehabilitation intervention is required.^[9,10]^ Traditional training methods are often simple in form, which is easy to cause the problem that children are difficult to adhere to long-term training. According to the research results of Amara K et al, the training shedding rate of the traditional group was 41%, which was significantly higher than 17% of the VR group (χ^2^ = 6.32, *P* = .01), and VR technology may improve this problem through immersive experience.^[11]^ Studies have revealed that immersive technology improves the physical and interpersonal abilities of children with ASD. For example, Lorenzo G G et al found that VR training could extend the duration of eye contact by 200 ms (95% confidence interval (CI): 150–250).^[12]^ However, the existing researches pay little attention to the indirect effect of parental evaluation on the rehabilitation effect, and the research on the mechanism of brain function is insufficient. To investigate the impact of VR treatment on motor balance, a retrospective analysis method was used. social ability and brain activation of children with ASD, as well as the degree of training completion and guardian.

## 2. Research subjects and methods

### 2.1. Research subjects

This research was approved by the Ethics Committee of Affiliated People’s Hospital of Hubei Medical College (Approval No. 2023-HPH-027). We retrospectively analyzed clinical data of 80 children with ASD who received either VR rehabilitation training or traditional rehabilitation training at our hospital between January 2024 and January 2025, all of whom met the Diagnostic and Statistical Manual of Mental Disorders, Fifth Edition diagnostic criteria. For analysis, children were stratified by symptom severity into high (n = 40) and low (n = 40) subgroups based on clinical records. Within each stratum, patients were classified according to the type of rehabilitation they had received (VR vs conventional). Inclusion criteria: 7 to 12 years old; confirmed diagnosis of ASD; presence of significant motor or social deficits; and availability of complete clinical data, including motor function, social behavior and brain imaging data. Exclusion criteria: severe physical diseases, such as congenital heart disease, severe epilepsy, etc; comorbidities with other psychiatric disorders; severe impairment of audiovisual function; and lack of key data in clinical data.

### 2.2. Research methods

A retrospective analysis of rehabilitation training programs was conducted based on case data. The experimental group underwent VR rehabilitation system intervention, training 5 times per week, 30 minutes per session, for 12 weeks. The VR rehabilitation system modules are shown in Table 1.

**Table 1:** VR rehabilitation training system modules and tasks.

Training module	Virtual task
Motor coordination training	Simulated walking (10 m round trip), real-time obstacle and speed adjustments
	Simulated balance exercises on stepping stones, balance beams, and floating platforms
	Object grasping or throwing tasks based on shape/color instructions
Social interaction	Simulated supermarket shopping, virtual classroom, and peer interaction tasks
Brain activation	Simulated memory-matching games
Guidance and feedback	Voice instructions, visual guidance, and point-based reward mechanisms

VR = virtual reality.

Each session consisted of randomized task combinations from these 4 modules, with training progress and difficulty adjusted based on children’s performance. The control group received traditional rehabilitation training, including: Motor coordination training: Gait correction, strength training, and joint mobility exercises, using repetitive motion training to promote neuromuscular recovery; motor games such as gripping, throwing, and puzzle-solving to enhance coordination; Social interaction training: Role-playing with therapists in simulated supermarket shopping, classroom discussions, and peer games to improve communication skills. Interactive activities such as board games and puzzles were used to enhance social engagement. Training frequency and duration matched the experimental group. Evaluation data were collected at 4 time points: before training (T0), after 6 weeks (T1), after 12 weeks (T2), and one month post-intervention follow-up (T3). The hospital’s ethics committee supported the study, and participants and their guardians provided informed consent. All data were anonymized to protect patient privacy.

### 2.3. Outcome measures

Objective and subjective evaluation metrics were extracted from case data, including: Subjective assessments: Test of gross motor development – second edition (TGMD-2),^[13]^ a scale that includes scores for 2 major skill dimensions, mobility and object control, continues to be scored based on the completion of the key motor elements of each skill, with 1 point awarded for normal execution of each element and 0 points awarded for failure to meet the Each element was scored as one point for normal execution and zero points for failure to achieve the key elements, with higher scores representing higher large muscle motor skills; autism treatment evaluation checklist (ATEC): A 77-item scale assessing ASD symptom improvement post-rehabilitation, with lower scores indicating better outcomes^[14]^; Psychoeducational Profile (3rd Edition): Evaluates ASD behavior improvement across communication, motor skills, social interaction, and cognitive adaptability, scored from 0 (unable) to 2 (fully independent).^[15,16]^ For clinical interpretation, previous studies suggest that a reduction of at least 25% in the ATEC total score, or approximately 10 points, is considered to reflect meaningful improvement in children with ASD^[15,16]^; Objective assessments: Table 2 details the measurement methods for motor coordination and social interaction abilities.

**Table 2:** Measurement methods for objective indicators.

Indicator	Measurement method	Instrument
Walking speed (m/s)	10 m walking time calculation. The faster the speed, the better coordination of the patient.	GaitRite pressure walkway
Gait stability	Step cycle, width, and length analysis. The lower the coefficient of variation, the more stable the gait.	Xsens IMU sensors
Trajectory change rate (°/s)	Record the rate of change of center of gravity or foot trajectory Angle during walking. The lower the change rate, the more stable the walking path.	
Head attitude change (°)	Open CV monitors head angle changes during conversations. Change Angle small, improve social attention more focused.	Vision sensors with open CV
Social speech frequency (times/min)	Record the number of active voice interactions in social scenarios. More communication means more patient engagement.	LENA Pro speech analysis
Brain activation (Beta)	Brain region activation measurement. The low activity in Beta band means that the patient has insufficient motor control.	Functional near-infrared spectroscopy (fNIRS)

Through case records and follow-up data, information on children’s and parents’ adherence and acceptance of the training was extracted: VR training acceptance: A Likert 5-point scale was used to assess children’s interest, ease of use, and sense of immersion in VR training. Scores ranged from 1 (strongly dislike/very difficult/no immersion) to 5 (strongly like/very easy/fully immersive). Researchers conducted face-to-face interviews with children using a simple questionnaire; Satisfaction survey: After completing the training, researchers used the system usability scale (SUS) to evaluate parents’ satisfaction with the rehabilitation training. The questionnaire consisted of 20 items, each scored on a scale of 1 (extremely satisfied) to 5 (highly dissatisfied), for a full score of 1 to 100. An improved overall score claimed that parents were more content with the VR teaching method. Acceptance was categorized into 3 levels: scores below 50 indicated low satisfaction, scores between 50 and 75 represented moderate satisfaction, and scores of 75 or above indicated high satisfaction. The questionnaire covered aspects such as ease of use, learning cost, and convenience, with sample questions including “I find this system complicated to use” and “I would let my child use this system again.”

### 2.4. Data analysis

All data were analyzed using SPSS 26.0 statistical software. The *t*-test was used to look at continuous factors, and the chi-square test (χ^2^) was used to look at categorical data. For brain activation data, paired *t*-tests were used to compare pre- and post-training brain activation in the high-score and low-score groups. Pearson correlation analysis was performed to calculate the correlation coefficient (*r*) between parental satisfaction and children’s training completion rate. A linear regression analysis was conducted to assess the effect of VR training on motor coordination and social interaction, adjusting for baseline variables to calculate the regression coefficient (β) and CI. Statistical analyses employed two-tailed tests, considering *P* < .05 as the threshold for significance.

As this was a retrospective study, no a priori sample size calculation was performed. However, a post hoc power analysis based on the between-group difference in TGMD-2 scores indicated a power of 0.87 (α = 0.05), suggesting that the current sample size of 80 participants was adequate to detect clinically meaningful effects.

## 3. Results

### 3.1. Participants

Before comparing the experimental and control groups, the baseline differences between the high-score and low-score groups were analyzed, as shown in Table 3.

**Table 3:** A comparison of basic attributes between higher and low-score groups.

Variable	High-score group	Low-score group	*P*-value
Age (yr)	8.9 ± 1.5	9.1 ± 1.6	.68
Gender (male/female)	32/8	30/10	.74
IQ test	98.5 ± 10.2	85.3 ± 9.8	.002
Language ability (PPVT/4)	89.2 ± 8.4	72.5 ± 7.6	.001
Motor coordination (TGMD-2)	30.4 ± 4.5	22.8 ± 3.9	.004

IQ = intelligence quotient, PPVT = Peabody Picture Vocabulary Test, TGMD-2 = test of gross motor development, second edition.

Table 3 shows no substantial variations in age as well as gender, comparing them both groups (*P* > .05), indicating comparability in demographic characteristics. However, the high-score group showed considerably greater intelligence quotient, verbal ability, motor coordination, and social competence than the low-score category (*P* < .05). These findings illustrate that VR training could produce a stronger intervention effect on children with poor scores. Table 4 presents the contrast of initial traits in the experimental and control groups.

**Table 4:** Comparison of baseline characteristics between experimental and control groups (mean ± SD).

Variable	Experimental group	Control group	Statistical value	*P*-value
Age (yr)	9.1 ± 1.5	8.9 ± 1.6	*t* = 0.58	.564
Male (%)	27 (67.5%)	29 (72.5%)	χ^2^ = 0.22	.637
ATEC total score	65.3 ± 10.2	66.1 ± 9.8	*t* = −0.35	.728
TGMD-2 score	42.7 ± 6.5	43.2 ± 6.3	*t* = −0.38	.705

ATEC = autism treatment evaluation checklist, TGMD-2 = test of gross motor development, second edition.

Table 4 shows that there were not any noteworthy distinctions throughout the 2 sets in terms of age, gender distribution, autism severity (ATEC score), or motor coordination (TGMD-2 score) (*P* > .05), confirming that the baseline characteristics of the 2 groups were comparable.

### 3.2. Comparison of motor coordination ability between groups

**Figure 1.**
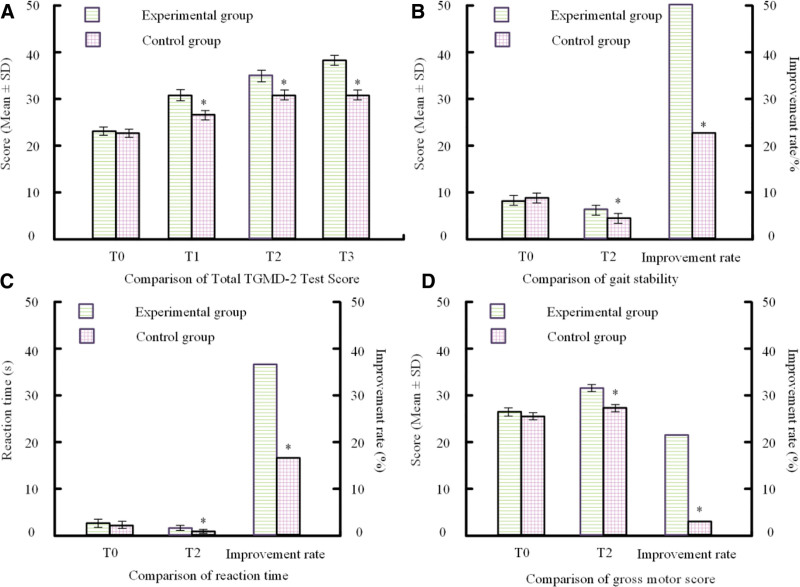
Comparison of TGMD-2 scores between groups. (A) Total TGMD-2 scores across 4 time points (T0, T1, T2, T3). (B) Gait stability at T0 and T2 and corresponding improvement rate. (C) Reaction time at T0 and T2 and improvement rate. (D) Gross motor scores and improvement rate. * *P* < .05 compared with the test group. TGMD-2 = test of gross motor development, second edition.

The TGMD-2 test was utilized to evaluate motor coordination in both groups of participants at four-point intervals (T0, T1, T2, T3). Figure 1 displays the intra-group comparative results.

Figure 1A demonstrates that all TGMD-2 scores of the the control and experimental sets at T0 were 22.3 ± 0.2 and 21.3 ± 0.5, respectively. However, at week 6 (T1) and week 12 (T2), the trial group had considerably higher scores (*P* < .05). At the 1-month follow-up (T3), the trial group maintained much greater TGMD-2 scores than the control group (*P* < .05). Additionally, Figure 1B reveals that the treatment group had considerably stronger gait stability than the controlling put (*P* < .05). Figure 1C indicates that reaction time also improved significantly comparable to the baseline category (*P* < .05). Figure 1D demonstrates that gross motor scores in the experimental group improved significantly (*P* < .05).

### 3.3. Effect comparison of VR rehabilitation training for children with different degrees of autism

**Figure 2.**
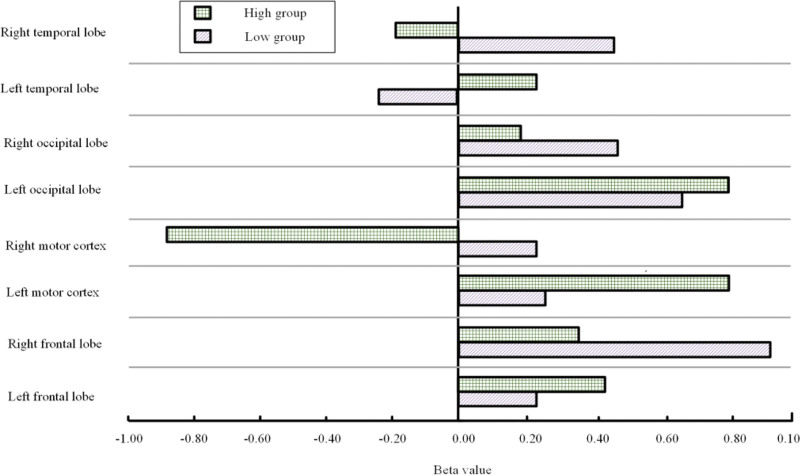
Brain activation after VR rehabilitation training in both groups. VR = virtual reality.

Figure 2 depicts the use of brain activation as an evaluation metric to compare all the advantages of VR rehabilitation training in the high-score and low-score groups.

Figure 2 shows that most brain regions exhibited positive activation during rehabilitation training in both groups. In the high-score group, all brain regions except for the right temporal lobe and right motor cortex (β values: −0.18 and −0.88) showed positive activation. In the low-score group, only the left temporal lobe (β value: −0.22) exhibited negative activation, while all other brain regions were positively activated. The study further explored the impacts of VR rehabilitation retraining on physical cooperation and social interaction abilities, as shown in Table 5.

**Table 5:** Comparison of motor coordination and social interaction abilities between the 2 groups (mean ± SD).

Category	Indicator	Low-score group (pre-training)	Low-score group (post-training)	*P*-value	High-score group (pre-training)	High-score group (post-training)	*P*-value
Motor coordination	Walking speed (m/s)	40 ± 0.08	0.55 ± 0.10	.018*	0.45 ± 0.09	0.75 ± 0.12	.005**
	Gait stability score	8.5 ± 1.2	6.0 ± 1.0	.021*	9.0 ± 1.3	4.2 ± 0.8	.002**
	Trajectory variation rate (%/s)	35 ± 5.3	25 ± 4.2	.015*	38 ± 6.1	15 ± 3.3	.001**
Social interaction	Social verbal communication frequency (times/min)	2.0 ± 0.5	3.5 ± 0.7	.034*	2.5 ± 0.6	6.0 ± 1.3	<.001***
	Head posture stability (°)	30 ± 4.2	18 ± 3.5	.027*	28 ± 5.1	10 ± 2.5	.003**

* Signals a notable distinction pre- and post-training (*P* < .05).

** Marks a markedly substantial variation (*P* < .01).

*** Reflects an exceptionally severe disparity (*P* < .001).

As shown in Table 5, after the rehabilitation training, the walking speed of the children in the high group increased from 0.45 ± 0.09 to 0.75 ± 0.12, and the gait stability score decreased from 9.0 ± 1.3 to 4.2 ± 0.8, and the rate of change of the trajectory was reduced by about 13 points, which was a significant difference (*P* < .05). The training effect was more significant in the lower group of children (*P* < .01). The high-score group showed a significant increase in social verbal communication frequency (2.5 ± 0.6 vs 6.0 ± 1.3, *P* < .001) and a notable improvement in head posture stability (28 ± 5.1 vs 10 ± 2.5, *P* < .01), indicating greater concentration during social interactions post-training.

### 3.4. Comparison of therapeutic effects on ASD

To determine the efficacy of VR treatment for children using ASD, the ATEC scale was used to assess and compare the pre- and post-rehabilitation scores of the experimental and control groups. Table 6 presents the scores of both groups in speech communication, social skills, sensory perception, health/behavior, and the total ATEC score before and after training.

**Table 6:** Comparison of ATEC scores between experimental and control groups for VR rehabilitation (mean ± SD).

Dimension	Experimental group (pre-training)	Experimental group (post-training)	*P*-value	Control group (pre-training)	Control group (post-training)	*P*-value
Speech communication (0–28)	20.2 ± 3.2	12.8 ± 2.8	.004**	21.5 ± 3.2	16.7 ± 3.5	.041*
Social skills (0–40)	26.1 ± 4.5	14.5 ± 3.7	.002**	27.8 ± 4.2	20.5 ± 4.7	.038*
Sensory perception (0–36)	18.2 ± 2.9	10.8 ± 2.5	.007**	17.9 ± 3.1	13.7 ± 2.9	.045*
Health/behavior (0–75)	42.1 ± 6.3	25.4 ± 5.8	.003**	41.3 ± 6.3	30.7 ± 6.1	.049*
Total ATEC score (0–179)	107.1 ± 8.2	69.7 ± 7.5	<.001***	108.9 ± 8.2	80.4 ± 7.8	.035*

ATEC = autism treatment evaluation checklist, VR = virtual reality.

* A substantial variance early and afterwards (*P* < .05).

** A notably major shift (*P* < .01).

*** Indicates an exceptionally noteworthy variance (*P* < .001).

As shown in Table 6, after VR rehabilitation training, the total ATEC score of the experimental group significantly decreased to 69.7 ± 7.5 (*P* < .01). Similarly, the comparison group’s ATEC dimension scores were significantly lower than before training (*P* < .01). To confirm the efficacy of VR rehabilitation treating ASD children, an individualized study of Psychoeducational Profile, Third Edition scale scores before and after training in both sets was performed, displayed in Table 7.

**Table 7:** Comparison of PEP-3 scores of VR rehabilitation training in experimental and control groups (mean ± SD).

Dimension	Experimental group (pre-training)	Experimental group (post-training)	*P*-value	Control group (pre-training)	Control group (post-training)	*P*-value
Communication ability (0–2)	0.82 ± 0.15	1.55 ± 0.20	.002**	0.80 ± 0.16	0.95 ± 0.18	.045*
Motor ability (0–2)	1.05 ± 0.18	1.72 ± 0.22	.004**	1.03 ± 0.19	1.15 ± 0.20	.062
Social ability (0–2)	0.75 ± 0.14	1.65 ± 0.19	.001**	0.74 ± 0.15	0.90 ± 0.17	.038*
Cognitive adaptive ability (0–2)	0.90 ± 0.17	1.80 ± 0.21	<.001***	0.88 ± 0.16	1.10 ± 0.19	.029*

PEP-3 = Psychoeducational Profile, Third edition, VR = virtual reality.

* Represents a obvious distinction between pre- and post-training (*P* < .05).

** Denotes a markedly significant variation (*P* < .01).

*** Highlights a profoundly significant distinction (*P* < .001).

In Table 7, the experimental group showed a highly significant improvement (*P* < .01) in communication ability, motor ability, social ability, and cognitive adaptive ability after VR rehabilitation training compared to before training. In the control group, post-training scores for communication ability, social ability, and cognitive adaptive ability also showed significant improvement (*P* < .05), while motor ability did not show a noticeable difference (*P* > .05).

### 3.5. Compliance and acceptability analysis of patients and guardians

The acceptance of VR rehabilitation by ASD children was evaluated at 5 time points, and the task completion rate at each stage was recorded to assess compliance. The results are shown in Table 8.

**Table 8:** VR training acceptability scores and task completion rate (Likert 5-point scale & percentage).

Evaluation dimension	T0	T1	T2	T3	T4
Interest score	2.1 ± 0.5	3.8 ± 0.6	4.2 ± 0.5	4.0 ± 0.4	3.9 ± 0.5
Ease of use score	2.5 ± 0.6	3.9 ± 0.5	4.3 ± 0.4	4.1 ± 0.2	4.0 ± 0.4
Immersion score	2.0 ± 0.5	3.7 ± 0.6	4.1 ± 0.5	4.0 ± 0.3	3.9 ± 0.6
Task completion rate (%)	40.2	65.8	82.3	78.2	75.3

VR = virtual reality.

Table 8 shows that ASD children’s acceptance of VR rehabilitation training increased significantly after 6 weeks of training (T1) compared to pre-training (T0), reaching the highest scores during the maintenance phase (Interest: 4.2, Ease of Use: 4.3, Immersion: 4.1). At T3 and T4, the scores declined slightly by an average of 0.17 and 0.27, but remained above 3.8. Additionally, task completion rate increased over time, peaking at 82.3% at T2, with a slight decrease at T3 and T4 but still remaining above 75%.

**Figure 3.**
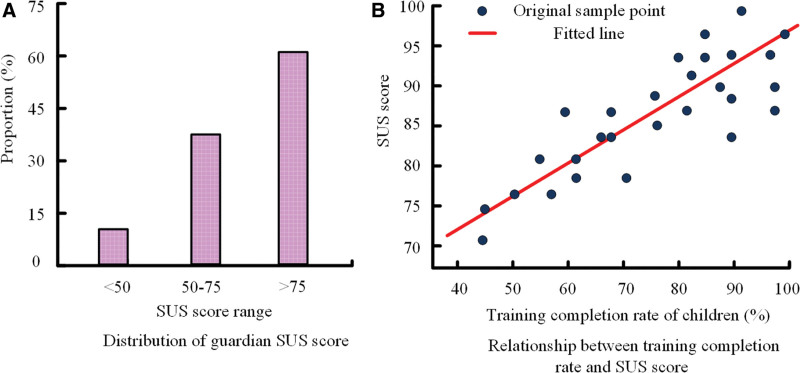
ASD guardian satisfaction analysis. (A) Distribution of guardian SUS score categories (<50, 50–75, >75) and corresponding proportion of respondents. (B) regression equation: SUS = 0.52 × (completion rate) + constant; correlation coefficient: *R* = 0.68, *P* < .01; regression coefficients: β = 0.52, 95% CI = 3.8–6.8. ASD = autism spectrum disorder, CI = confidence interval, SUS = system usability scale.

Figure 3A shows guardian satisfaction scores, with 53% of guardians being highly satisfied with VR rehabilitation training. Figure 3B further reveals a significant positive correlation between guardian satisfaction and children’s training completion rate (*R* = 0.68, *P* < .01). The regression analysis indicates that for every 10% increase in completion rate, the SUS score rises by 5.2 points (β = 0.52, 95% CI: 3.8–6.6, *P* < .01). When children’s completion rate exceeded 80%, most guardians’ SUS scores were above 78; for children with completion rates below 50%, SUS scores were mostly under 60. These findings indicate that high compliance with VR training significantly enhances parental recognition of the system, further supporting its effectiveness and acceptability.

## 4. Discussion

Children with ASD often face persistent difficulties in motor coordination and social interaction. Conventional rehabilitation, while widely applied, is limited by its low interactivity and often results in poor treatment adherence.^[17]^ In contrast, VR technology provides an immersive and engaging environment that encourages active participation. By integrating visual, auditory, and proprioceptive feedback, VR has the potential to enhance neuroplasticity and sustain children’s interest in long-term rehabilitation.^[18]^ These features make VR particularly suitable for pediatric populations, where motivation and compliance are critical for therapeutic success.

Beyond behavioral improvement, the present findings add to growing evidence that VR rehabilitation may also influence brain function. Functional near-infrared spectroscopy (fNIRS) data indicated activation in regions involved in motor planning, imitation, and social cognition, consistent with the “motor model activation brain region” theory.^[19–21]^ Specifically, enhanced activation in the prefrontal cortex and parietal lobe is noteworthy, as these areas are associated with executive control, attention regulation, and sensorimotor integration – domains frequently impaired in ASD. Increased premotor and mirror neuron–related activity suggests that VR may facilitate imitation and action observation learning, processes crucial for both motor and social development. By contrast, reduced activation in temporal regions observed in some children could reflect persistent challenges in language and socio-emotional processing, consistent with previous neuroimaging findings in ASD.^[22]^ Together, these results suggest that VR training does not merely improve behavior, but may also engage compensatory neural mechanisms that underpin motor coordination and social interaction. Importantly, children with lower baseline abilities demonstrated more pronounced neural and behavioral gains, underscoring the potential of VR for precision rehabilitation tailored to individual needs.

The study also highlights the role of caregivers in rehabilitation outcomes. Higher training adherence was closely linked to greater caregiver satisfaction, suggesting that VR systems not only benefit children but also foster parental acceptance of therapy. This supports the notion that family engagement is an essential component of successful intervention programs. Previous research has primarily examined parental attitudes in isolation,^[23,24]^ but the current study extends this by demonstrating a direct relationship between children’s compliance and caregiver recognition. This emphasizes the dual benefit of VR: enhancing child performance while reinforcing family support.

Clinically, these findings suggest that VR-based rehabilitation could serve as a promising adjunct or alternative to traditional therapy. Its capacity to improve both motor and social domains, while simultaneously engaging families, makes it a valuable tool for personalized pediatric neurorehabilitation.^[25–27]^ Nevertheless, challenges remain. The durability of VR-induced benefits over time, the optimal training intensity, and the integration of VR into routine clinical workflows warrant further investigation. Additionally, while fNIRS provided useful insights, future studies should incorporate multimodal approaches such as electroencephalography–fNIRS to capture more comprehensive patterns of neural adaptation.

Several limitations should be acknowledged. First, participants were recruited from a single tertiary hospital in Hubei Province, which may restrict the generalizability of the findings to other regions or healthcare systems. The sample therefore may not fully represent children with ASD from diverse cultural, educational, and socioeconomic backgrounds, factors that are likely to influence rehabilitation engagement and caregiver attitudes. Second, as a retrospective study, no prospective sample size calculation was performed; although post hoc analysis indicated adequate power, the modest sample size still limits the robustness of subgroup analyses. Third, brain activation was evaluated solely with fNIRS, which primarily measures cortical surface hemodynamics. This single-modality approach cannot capture deeper brain structures or provide the temporal precision of methods such as electroencephalography, potentially leading to incomplete interpretation of neural mechanisms. Fourth, the retrospective design may introduce selection and information biases, as data relied on existing medical records and caregiver reports. Finally, training adherence and satisfaction were assessed using self-report scales, which are susceptible to reporting bias. Future multicenter, prospective trials with larger and more heterogeneous samples, combined with multimodal neuroimaging and objective behavioral assessments, are warranted to validate and extend these findings.

In conclusion, VR rehabilitation offers more than short-term performance gains: it provides a pathway to sustained motor and social development, supported by both functional neuroscience evidence and caregiver acceptance. By bridging neural mechanisms with clinical outcomes, VR can contribute to more individualized and family-centered approaches in ASD rehabilitation.

## Author contributions

**Conceptualization:** Hui Yi, Xiufang Zhou, Shengju Quan, Tianyan Liu.

**Data curation:** Hui Yi, Xiufang Zhou, Shengju Quan, Tianyan Liu.

**Formal analysis:** Hui Yi, Xiufang Zhou, Tianyan Liu.

**Investigation:** Xiufang Zhou, Shengju Quan, Tianyan Liu.

**Methodology:** Hui Yi, Shengju Quan.

**Software:** Tianyan Liu.

**Supervision:** Shengju Quan, Tianyan Liu.

**Validation:** Xiufang Zhou, Shengju Quan, Tianyan Liu.

**Visualization:** Hui Yi, Xiufang Zhou, Shengju Quan, Tianyan Liu.

**Writing – original draft:** Hui Yi, Xiufang Zhou, Shengju Quan, Tianyan Liu.

**Writing – review & editing:** Hui Yi, Xiufang Zhou, Shengju Quan, Tianyan Liu.
